# High-contrast enzymatic immunohistochemistry of pigmented tissues

**DOI:** 10.14440/jbm.2016.122

**Published:** 2016-07-12

**Authors:** Sara M. Duncan, Gail M. Seigel

**Affiliations:** University at Buffalo, Center for Hearing & Deafness and SUNY Eye Institute, Buffalo, NY, USA

**Keywords:** enzyme immunohistochemistry, pigmented tissue, peroxidase substrate,VIP purple substrate, diaminobenzidine, retinal pigmented epithelial cells

## Abstract

Historically, standard enzyme immunohistochemistry has been accomplished with brown (DAB, diaminobenzidine) substrate. This can become problematic in pigmented tissues, such as the retina, where brown pigment of retinal pigmented epithelial (RPE) cells can be easily confounded with brown substrate. Although immunofluorescence detection methods can overcome this challenge, fluorescence may fade over a period of weeks, while enzyme substrates allow for more long-lasting, archival results. In this report, we will describe a high-contrast enzyme immunohistochemistry method ideal for pigmented tissues that utilizes purple (VIP) substrate. We compared brown (DAB) and purple (VIP) substrates in enzyme immunohistochemistry experiments using human retina (paraffin sections) and monkey retinal pigmented epithelial cells (frozen sections), both containing brown pigmented cells. We compared substrates using several primary antibodies against markers that can be detected in the retina, including GFAP, VEGF, CD147 (EMMPRIN), RHO (rhodopsin) and PAX6. Methyl green was used as a counterstain for paraffin sections. A side-by-side comparison between DAB and VIP immunohistochemistry showed excellent contrast between pigmented cells and the purple VIP substrate in both human retinal tissue and monkey pigmented epithelial cells for all of the markers tested. This was a marked improvement over DAB staining in pigmented cells and tissues. For both paraffin sections and frozen sections of pigmented tissues, purple VIP substrate is an excellent alternative to brown DAB substrate and non-permanent immunofluorescence methods.

## BACKGROUND

Immunohistochemistry is an important diagnostic and experimental tool used for the identification and localization of cell and tissue components. Early immunostaining methods began in 1930’s, with improvements in sensitivity with the introduction of enzymatic methods in the 1960’s [1]. Subsequently, the development of diaminobenzidine molecule (DAB) as an enzymatic substrate led to a stable brown reaction product, more permanent than fluorescent methods. Currently, DAB is the most widely used chromogen [1] and remains the gold standard in enzymatic immunohistochemistry. However, this brown DAB reaction product can be difficult to distinguish from surrounding pigment granules commonly seen in retinal pigmented epithelial (RPE) cells, melanocytes, macrophages, lymphoid aggregates of human gut associated lymphoid tissue (GALT) and hepatocytes.

Several methods to address the challenge of enzymatic staining in pigmented tissues have been employed, such as tissue bleaching [2, 3] (sometimes with damaging effects on the tissue being examined), as well as less permanent fluorescent methods. Here, we chose an alternative peroxidase substrate from among a few non-brown options: purple (VIP), red (NovaRED) and blue (SG) (Vector Labs, Burlingame, CA). We chose the bright purple VIP substrate that would provide the most contrast against the brown pigment of RPE cells. Red substrates, such as NovaRED and 3-amino-9-ethylcarbazole (AEC) were considered, but based on previous experiments did not always show good contrast in brown pigmented tissue, which can sometimes exhibit a reddish cast. The VIP purple substrate has been around for a number of years, used in a few studies involving pigmented tissues [4, 5, 6], but never presented as a stand-alone methodology or directly compared with DAB in pigmented tissues. Here, we present an immunohistological comparison of DAB and VIP as enzyme substrates for pigmented cells and tissues of the retina, with applications for all pigment-containing tissues.

## MATERIALS AND METHODS

Archival de-identified paraffin sections of human eyes with retinal degenerative disease were obtained with University Institutional Review Board approval. We purposely chose diseased eyes instead of healthy eyes so that our specific markers of interest would be most prominent. Monkey RPE cells were purchased from Lonza (Walkersville, MD), grown in culture on filter inserts, processed and sectioned into frozen sections as provided by Dr. Bruce Pfeffer and Dr. Steven Fliesler (see acknowledgements). Third passage rhesus macaque retinal pigment epithelial (RPE) cells (derived from primary cultures prepared in accordance with ARVO guidelines) were grown on a Millicell insert (fabricated using tissue culture treated Isopore^TM^ polycarbonate, track-etched with 3-µm pore size, 12 mm diameter; EMD-Millipore, Billerica, MA) coated with mouse EHS tumor laminin from Sigma-Aldrich (a1b1g1, or “laminin 111”), at 10 micrograms/cm^2^ (Sigma-Aldrich, Saint Louis, MO). Pertinent details of the culture medium include reduction of the serum component to 1% (v/v) bovine calf serum, addition of defined components including transferrin, IGF-1 peptide fragment, trace elements and nutrients, hormones, and supplementation with an aqueous-soluble extract of bovine neural retinal tissue. 50,000 cells were plated in the device, and upon confluence in “low” calcium medium ([Ca^++^] below 0.1 mM), the culture was switched to medium containing [Ca^++^] at 0.5 mM, and maintained for 6 months as a stable monolayer that exhibited a characteristic cobblestone morphology as well as development of melanin pigmentation [7]. Under similar growth conditions, these cells previously were shown to express RPE cell markers, including cellular retinaldehyde-binding protein [8], and pigment epithelium-derived factor [9], all indications of RPE differentiation and suitability for our studies of RPE markers expressed *in vitro*.

RPE cells were fixed in room temperature 4% formaldehyde (from 37% stock solution, Sigma) in HEPES-buffered modified Hanks’ balanced salt solution [10]. The inserts were incubated in a graded sucrose series (10, 20, and 30% [w/v] in PBS) and held overnight in a 2:1 mixture of OCT Embedding Compound (Andwin, Schaumburg, IL) and 30% sucrose. The polycarbonate filter was detached from its housing, frozen in OCT in a plastic mold, and stored at –80°C until sectioning. 12–16 µm transverse sections through both the cell layer and the polycarbonate filter material were taken using a Leica CM 3050S cryotome at a chamber temperature of –20°C, and affixed to Superfrost slides (Electron Microscopy Sciences, Hatfield, PA), which were stored at –20°C until staining.

The primary antibodies, sources and concentrations that were used in these experiments are listed in **Table 1**. Markers were chosen to highlight both pigmented RPE cells (CD147, VEGF, PAX6), as well as adjacent retinal cells, such as photoreceptors (rhodopsin) and glial cells (GFAP). All antibodies were prepared using a 1:100 dilution with PBS-Tween. Scytek CRF polymer kit (Scytek, Logan, UT) was used for blocking, polymer and DAB substrate incubations. Purple VIP substrate was obtained from Vector Laboratories (Burlingame, CA) and used instead of DAB for comparison purposes.

**Table 1.** Antibodies used in this study.

Marker	Company Info, Catalog Number	Type	Concentration
CD147 (EMMPRIN)	Pharmingen BD, 555961	Mouse IgG1	5.0 μg/ml
Rhodopsin (RET-P1)	Chemicon, MAB5316	Mouse IgG1	0.2 μg/ml
VEGF	Santa Cruz, SC7269	Mouse IgG2a	0.2 μg/ml
GFAP	Cell Signaling Technologies, 3670P	Mouse IgG1	N/A*
PAX6	Abcam, Ab5790	Rabbit Poly	10 μg/ml

^*^N/A, Not provided by the manufacturer.

### Immunohistochemistry of paraffin sections of human eyes

Both VIP and DAB experiments began with deparaffinization: xylene for 8 min, then 100% ethyl alcohol, 95% ethyl alcohol, 70% ethyl alcohol, and water for 3 min each. The sections were boiled in a citrate antigen retrieval buffer (10 mM Sodium Citrate, 0.05% Tween 20, pH 6.0) for 25 min. Next, slides were cooled in PBS and the sections were encircled with a hydrophobic PAP pen. All incubations at this point forward were carried out at room temperature. The sections were permeabilized in PBS with 0.05% Tween (PBS-Tween) for 5 min. Scytek Peroxide Block was then applied for 5 min to block endogenous peroxide activity. Sections were rinsed three times in PBS and incubated in Scytek Super Block for 4 min. After three more rinses in PBS, the sections were incubated for 1 h with one of the antibodies listed in **Table 1**, or an isotype (negative) control antibody diluted in PBS-Tween.

At the end of the primary antibody incubation, the sections were rinsed 3 times in PBS, followed by a 30-min incubation in Scytek CRF Anti-Polyvalent HRP Polymer. The Scytek CRF Anti-Polyvalent Polymer is a proprietary, biotin-free formulation of an HRP-linked polymer that binds to primary antibodies of mouse, rat, rabbit and guinea pig origin with high sensitivity. After incubation with polymer, the slides were then rinsed 3 times in PBS and once in water. The final peroxidase substrate (DAB or VIP) was prepared according to manufacturers’ instructions and applied to the sections for 5 min. The sections were then rinsed in water for 5 min. For the methyl green counterstain, the slides were placed on a 60ºC warm platform and incubated in methyl green solution (0.5% methyl green in 0.1 M sodium acetate buffer, pH 4.2) for 5 min. The sections were blotted and briefly rinsed in water before the dehydration process. Each slide was dipped quickly 10 times in 95% alcohol and two changes of 100% alcohol. Lastly they were cleared in xylene and coverslips were applied using Permount mounting medium (Fisher Scientific, Waltham, MA). Images were captured using a Nikon ES600 microscope with a SONY ICX 285AL SPOT camera with Spot software.

**Figure 1:**
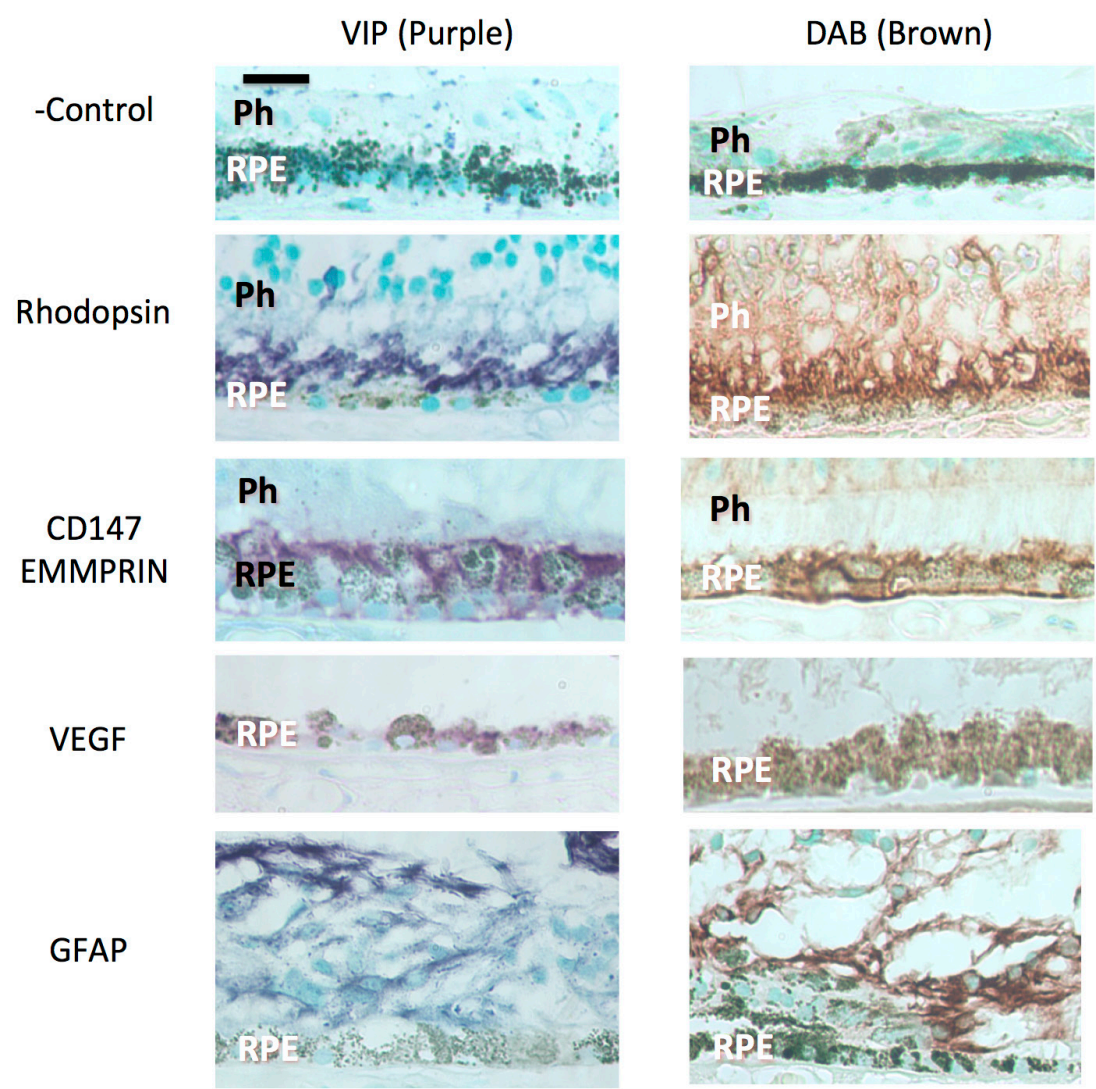
**VIP vs. DAB peroxidase substrates in human retinal sections**. Rhodopsin, EMMPRIN, GFAP and VEGF were examined in human retinal sections using VIP (left panels) and DAB (right panels) as per Materials and Methods. RPE indicates RPE layer, while Ph indicates photoreceptor layer. In the case of some images (*e.g.*, VEGF and GFAP), the photoreceptor layer is almost nonexistent due to retinal degeneration. Original magnification: 200 ×. Scale bar = 5 µm. Control, Isotype control

### Immunohistochemistry of frozen sections of pigmented monkey RPE cells

The monkey RPE sections were dried on the countertop overnight at room temperature and then permeabilized in PBS-Tween for 5 min, followed by Scytek Peroxide Block for 5 min. Sections were rinsed three times in PBS and incubated in Scytek Super Block for 4 min. After three rinses in PBS, the sections were incubated for 1 h with one of the antibodies listed in **Table 1**, or an isotype (negative) control antibody. After the 1 h incubation, the sections were rinsed 3 times in PBS, followed by a 30-min incubation in Scytek CRF Anti-Polyvalent HRP Polymer. After incubation with polymer, the slides were then rinsed 3 times in PBS and once in water. The peroxidase substrate (DAB or VIP) was applied to the sections for 5 min. The sections were then rinsed in water for 5 min and mounted with aqueous mounting medium. Images were captured using a Nikon ES600 microscope with a SONY ICX 285AL SPOT camera with Spot software.

## RESULTS

We began our substrate comparison with paraffin sections of human eyes. All of the human eyes in this study showed signs of severe retinal degeneration as evidenced by thinning of the retinal layers and gliosis. We chose markers for immunohistochemical analysis based upon anticipated expression by pigmented RPE cells themselves (EMMPRIN, VEGF) or in retinal cells adjacent to RPE cells, such as photoreceptors (rhodopsin) and glial processes (GFAP). In **Figure 1**, a side-by-side comparison of VIP vs. DAB substrates illustrates the robust purple reaction product of VIP in proximity to the pigmented RPE cells of the human retina. The purple VIP color was a much better contrast than the brown DAB counterpart for each of the four markers examined in human retinal tissues. It is important to note that as with many human tissues, there is naturally occurring variability in RPE pigmentation from one individual to another, evident in our own results.

**Figure 2:**
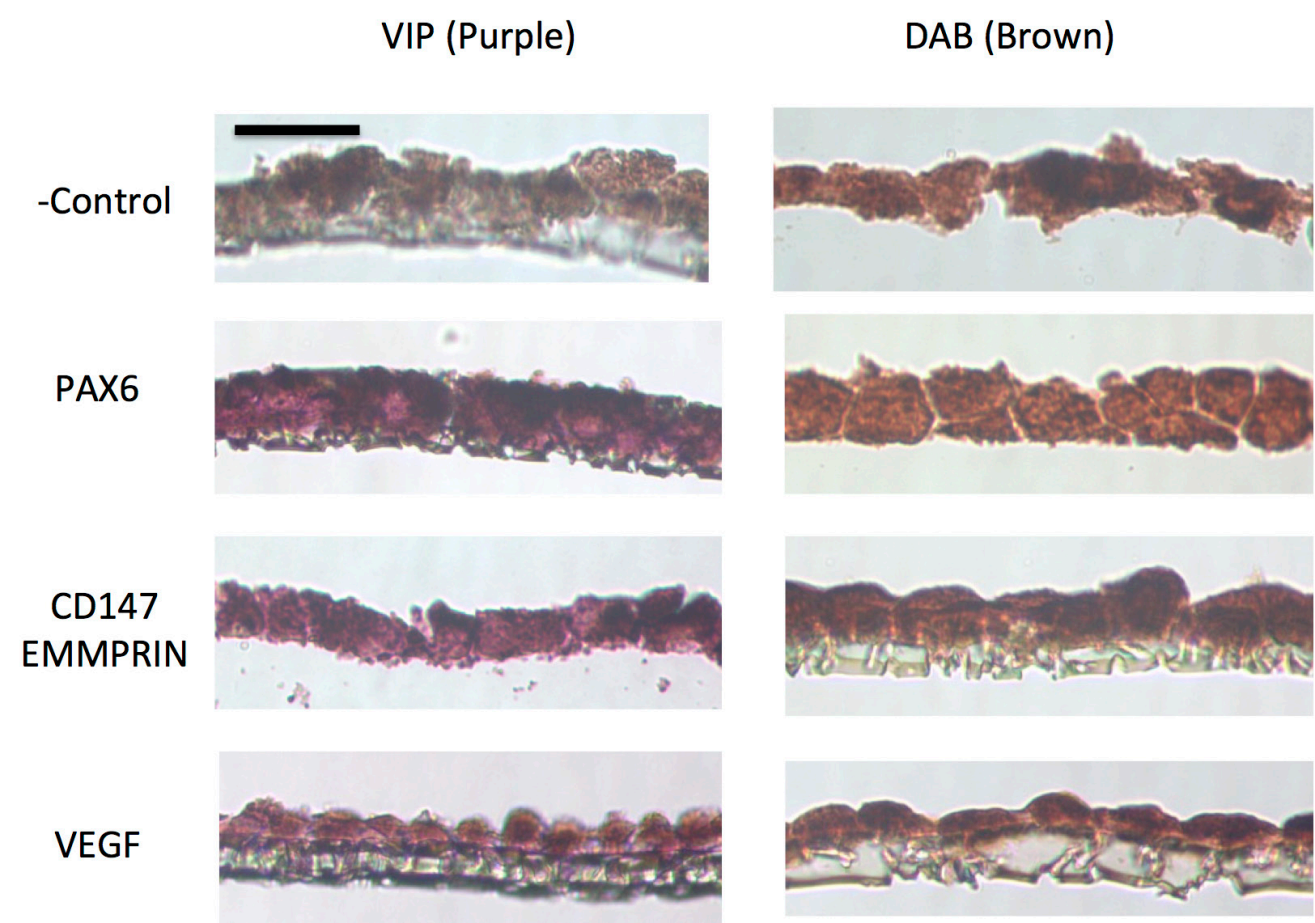
**VIP vs. DAB peroxidase substrates in monkey RPE cells**. PAX6, EMMPRIN and VEGF were examined in monkey RPE cells using both DAB (right panels) and VIP (left panels) as per Matherials and Methods. Original magnification: 200 ×. Scale bar = 5 µm.

We continued our substrate comparison with frozen sections of monkey RPE cell monolayers grown on filter inserts that exhibited darker pigmentation as compared with human RPE cells. Since these monkey RPE cell preparations contained only pigmented cells, we omitted the methyl green counterstain to focus on the substrate comparisons. We chose three markers that would be expressed in RPE cells, namely PAX6, VEGF and EMMPRIN. As evident in **Figure 2**, the VIP purple substrate is visible for all three markers despite the brown pigment inherent in the RPE cells. In contrast, it was much more difficult to distinguish between pigmented RPE cells and the brown DAB substrate for each of the corresponding markers.

## DISCUSSION

In this report, we describe a high contrast method for staining retinal tissues involving purple VIP substrate and a methyl green counterstain. VIP substrate is previously known for its usefulness in single and double marker enzyme immunohistochemistry [11, 12], with some applications in pigmented tissues [4, 5, 6]; but this method has not been widely implemented for pigmented tissues nor specifically described for this purpose as a Methods report.

Here we compared the standard brown DAB substrate with purple VIP substrate in peroxidase enzyme-based immunohistochemistry using pigmented cells and tissues from both human and monkey retina. We anticipated that if the pigmentation were too dark, even a bright substrate color would not be visible amid the pigment granules. However, in our experiments, we were able to see the purple substrate reaction quite clearly even in the most darkly pigmented cells in our study, the monkey RPE cells. This is an encouraging result; although the possibility remains that more darkly pigmented cells in other tissues could create more of a challenge.

We chose markers that would be expressed in RPE cells themselves (EMMPRIN, PAX6 and VEGF) or in cells adjacent to RPE cells (Rhodopsin, GFAP). The human retinal tissues that we chose for this study had all undergone severe retinal degenerative disease processes, which aided in the detection of some of our markers. For example, Vascular Endothelial Growth Factor (VEGF) is upregulated in macular degenerative disease (ARMD) [13, 14] and this was clearly seen in our VEGF immunohistochemical staining of retinal tissues from ARMD patient samples. Monkey RPE cells cultured on filter inserts were also immuno-positive for VEGF, not surprising due to the stresses of the cell culture environment. Gliosis is a common feature of retinal degenerative disease, characterized by enhanced expression of glial fibrillary acidic protein (GFAP) [15]. In our diseased human retinal tissues, we saw extensive GFAP immunoreactivity that corresponded with retinal atrophy. Rhodopsin, a naturally-occurring photoreceptor protein that has been implicated retinal degenerations [16, 17] is expressed adjacent to the RPE and was clearly visible in our human retinal sections. EMMPRIN is a well-established marker for RPE cells, with apical polarity of expression [18] and showed robust staining in both human and monkey RPE cells, particularly with the VIP substrate. PAX6 regulates melanogenesis in RPE cells [19] and was also expressed in cultured monkey RPE cells, most likely as a means to regulate melanin expression in an *ex vivo* environment. In all of the markers tested, the purple VIP substrate gave superior results as compared to its DAB counterpart for pigmented cells and tissues. Additional signal enhancement may also be possible in paraffin sections with the concomitant use of reagents such as citraconium anhydride as an antigen retrieval method that can aid in overcoming antigen-masking paraffin crosslinks [20]. Citraconium anhydride was not necessary in our experiments, but may be considered as an additional signal enhancement for harder-to-detect antigens.

In the event that VIP contrast falls short with very dark pigments, there are bleaching methods that can be used as alternatives, although they require very careful adjustment to prevent tissue damage. One fairly straightforward melanin bleaching method involves 10% hydrogen peroxide at 65ºC for 30 min [21], which was superior in retaining tissue morphology when compared with incubation of ocular tissues in potassium permanganate and oxalic acid [22]. In our hands, the VIP substrate performed well for a wide spectrum of RPE pigmentation, both *in vitro* and *in vivo* with no need for extra bleaching steps. The substrate incubation step, whether DAB or VIP, is a normal part of the experimental protocol, so the use of VIP added no extra incubation time to the experiment, another advantage over the existing bleaching methods.

## CONCLUSIONS

In this report, we show the advantage of using an enzymatic purple substrate (VIP) with a methyl green counterstain for improved contrast in tissues containing pigmented cells, such as the retina. The purple VIP/methyl green method is a viable and useful alternative to the standard DAB method for enzymatic immunohistochemistry in pigmented tissues.

## Abbreviations used

AEC: 3-amino-9-ethylcarbazole
ARVO: Association for Research in Vision and Ophthalmology
DAB: diaminobenzidine
GALT: gut-associated lymphoid tissue
GFAP: glial fibrillary acidic protein
HEPES: 4-(2-hydroxyethyl)-1-piperazineethanesulfonic acid
IGF-1: insulin-like growth factor-1
OCT: optimal cutting temperature
PBS: phosphate buffered saline
PAX6: paired box protein 6
RHO: Rhodopsin
VEGF: vascular endothelial growth factor
RPE: retinal pigmented epithelial
VIP: proprietary purple substrate
